# Interface Modification, Water Absorption Behaviour and Mechanical Properties of Injection Moulded Short Hemp Fiber-Reinforced Thermoplastic Composites

**DOI:** 10.3390/polym13101638

**Published:** 2021-05-18

**Authors:** João P. Manaia, Ana Manaia

**Affiliations:** Laboratory for Wear, Testing & Materials (LED&MAT), Instituto Pedro Nunes, Rua Pedro Nunes, 3030-199 Coimbra, Portugal; ana.manaia@ipn.pt

**Keywords:** hemp fibers, HDPE, PP, maleic anhydride, stearic acid, water absorption

## Abstract

The effects of maleic anhydride, stearic acid and water absorption on the physical and flexural properties of injection moulded short hemp fiber-reinforced thermoplastic composites were investigated, in order to understand the suitability of these materials for outdoor applications. The water absorption, diffusion mechanisms and kinetics of composites were evaluated by immersing the specimens in distilled water at 23 °C. Flexural fracture surface morphologies were investigated in order to compare the results of flexural tests with qualitative morphological observations. The process of water absorption was found to follow the Fickian mode of diffusion. Flexural properties ($E_{f}$ and $\sigma_{fm}$) were affected by the water absorption. The addition of maleic anhydride and stearic acid enhanced the resistance to water absorption of composites and resulted in a slight increase of flexural properties of composites based on a high-density polyethylene (HDPE) matrix. The reduction in flexural properties induced by the degradation of matrix-fiber interfacial bonding due to water absorbed was confirmed by scanning electron microscopy analysis.

## 1. Introduction

Natural fiber-reinforced polymer composites, produced by combining lignocellulosic fibers with phenolic resin, were first introduced in 1908. The use of hemp fibers as reinforcement in composites have been attracting scientific interest as a response to the challenges in developing environmentally friendly materials with a reduced carbon footprint as well as open up new application fields [1,2]. In 2019, according to AVK’s study on the European market for glass fiber-reinforced plastics, the total estimated production was 1.141 million tonnes. Glass fibers accounts for 90% of the total market volume for fiber-reinforced plastics (short and long fibers, roving, woven, fabrics and mats), whereas natural fibers account for only 2–2.5% and are currently being used in construction (decking), automotive (door trim panels, engine and transmission covers) and packaging applications [3]. Hemp fibers are one of the strongest bast fibers available, in which the predominant chemical components are carbohydrates (cellulose, hemicellulose and pectins) in combination with aromatic alcohols such as lignin. The structure of hemp fibers is characterized by a system of pores, capillaries, voids and central channel, named lumen [4]. Despite the better properties of hemp fibers, such as lower density (1.2–1.6 g/cm^3^) compared to that of glass fiber (2.4 g/cm^3^), high specific mechanical properties as well as relatively low cost, renewability and biodegradability, their inherent hydrophilic character, low resistance to micro-organisms, low thermal stability and flammability, restricts their use in many structural as well as outdoor applications, thus hindering its market growth [5,6,7,8]. Also, the structure and mechanical properties of hemp fibers depend on their physical, chemical and morphological properties as well growing conditions and harvesting time [9]. Hemp fibers are used to reinforce thermoplastics with the aim of increasing their strength [10]. The advantages of thermoplastics over thermoset matrices used in composites include the greater design freedom, as they are suitable for injection moulding and offer better recycling possibilities [9]. Polypropylene (PP) and polyethylene (PE) matrices are the most used in natural fiber composites, due to their lower processing temperatures, which are below to the decomposition temperatures of lignocellulose materials, better recyclability, lower density and cost and higher tensile strength, heat and corrosion resistance compared to other thermoplastics [11], although, the lack of interfacial adhesion of hydrophobic PP and PE with the hydrophilic lignocellulosic hemp fibers is a problem due to their different chemical structures [2,11]. PP and PE are non-polar hydrophobic polymers without any polar group, while hemp fibers have hydroxyl groups in their structure which tends to give active polar hydrophilic material. During composite processing the combination of both hydrophobic and hydrophilic materials results in poor matrix-fiber interfacial adhesion, due to insufficient wetting, leading to ineffective stress transfer [2]. Also, the poor hygrothermal resistance of natural fiber composites results in moisture absorption leading to fiber swelling, dilapidation, formation of cracks and debonding of the matrix-fiber interface, affecting the mechanical properties, lifetime and dimensional stability of the composites [12,13,14]. Over the years, a great number of studies have focused on increasing the matrix-fiber interfacial adhesion through physical [15] and chemical surface treatments [10,16] or adding coupling agents [2,11,13,15,16,17,18,19,20]. By adding a coupling agent, such as maleic anhydride, the resultant polymer is a polymer modified with a polar group in the chain, which is able to reacts with the hydroxyl groups (OH) on the fibers’ surface to form strong hydrogen bonds [9]. These bonds will effectively interlock the matrix with fiber promoting a better stress transfer between both [2], leading to a reduction of water absorption and increasing the mechanical properties of the composites [20]. The direct incorporation of coupling agents during composite compounding avoids the use of solvents as needed in chemical treatments, being thus more environmental-friendly. Wang et al. [21] studied the effect of maleic anhydride-grafted polyethylene on short hemp fiber-reinforced HDPE composites. The results showed that the dynamic compressive behaviour and flame retardancy of the maleic anhydride polyethylene-added composites were improved compared to those without the coupling agent. Stearic acid has also been used as a coupling agent to promote matrix-fiber dispersion and decrease the hydrophilicity of lignocellulosic fibers. The carboxyl group in stearic acid reacts with the hydroxyl groups of cellulose to form hydrogen bonds [22]. Kiattipanich et al. [23] investigated the effect of stearic acid on the mechanical properties of PP composites reinforced with sugarcane fibers. They showed that stearic acid-treated sugarcane fiber improved the mechanical properties of composites by increasing the fiber dispersion and the interfacial adhesion between the matrix and fiber. In addition, the stearic acid acted as a lubricant, reducing the shear viscosity and increasing the melt flow index. Throughout the years, several authors have been interested on the potential use of natural fibers in composite materials. Nevertheless, references on the effects of both maleic anhydride and stearic acid on the interfacial adhesion between the matrix and fiber and on the physical and mechanical properties of injection moulded short hemp fiber-reinforced thermoplastic composites, are not abundant in the literature. Also, the available relevant experimental data is not consistent and the existing results are not conducive to drawing any conclusions. A literature review focused on hemp fiber-reinforced polypropylene and polyethylene composites, based on injection mould manufacturing, is provided in Table 1 and Table 2, respectively, and summarized hereafter.

The purpose of the present research project was fivefold: (1) to investigate the rheological behaviour of composite formulations; (2) to provide a better understanding of the effect of different weight percentages of hemp fiber, as well as the effect of maleic anhydride and stearic acid, on the mechanical properties of both polypropylene and polyethylene injection-moulded short hemp fiber-reinforced composites; (3) to determine the influence of water absorption on the physical properties of composite materials and also, to analyse the water diffusion mechanism and kinetics as well as to compare the experimental diffusion results with theoretical predictions in order to find deviations from the regular Fickian mode; (4) to investigate the effect of water absorption on the flexural properties and (5) to compare the results of mechanical tests with qualitative morphological observations. The present paper is organized into four sections: Section 2 details the investigated materials, composite manufacturing and the mechanical testing protocols. The experimental results are displayed and discussed in Section 3. Some concluding remarks are finally given in Section 4.

## 2. Materials and Methods

### 2.1. Materials

ISPLEN^®^ PP 090 G2M, a polypropylene (PP) homopolymer and RIOPOL^®^ BI-56250, a high-density polyethylene (HDPE), obtained from Repsol (Portugal) and Riopol (Portugal), respectively, were used as the polymer matrix. Maleic anhydride Fusabond™ M603, provided by Safic Alcan (Maia, Portugal), was used as a coupling agent between hemp fibers and both polyolefin matrices (PP and HDPE). Acros Organics™ stearic acid with 97% purity, purchased from Fisher Scientific (Oeiras, Portugal), was used to promote the fiber dispersion in the matrix and overall composite processability as well as to improve the matrix-fiber compatibility. Short hemp fibers, with a nominal length between 6 and 10 mm, were supplied by Fibers Recherche Développement (frd, Troyes, France). Hemp fibers were extracted mechanically, the shives and dust content were lower than 5%. The technical information for ISPLEN^®^ PP 090 G2M [27], RIOPOL^®^ BI-56250 [28], Fusabond™ M603 [29], Acros Organics™ stearic acid 97% [30] and short hemp fibers [31] were taken from the supplier’s data sheets and are given in Table 3. Hereafter, these materials will be labelled PP, HDPE, CA, SA and HF, respectively.

### 2.2. Compound and Specimens Preparation

Composites based on PP and HDPE matrices, with two different weight fractions of HF: 20 wt% and 40 wt% (based on total composite weight), along with CA and SA, were prepared. The total weight percentage of CA and SA in the composites, were fixed at 3 wt%. All composite formulations prepared for this study are listed in Table 4.

The values of both, 40 wt% HF and 3 wt% CA and SA content in the composite formulations were based on literature according to the research conducted by Pickering et al. [16], in which composites with different weight percentages of HF, MAPP and PP were injection-moulded into tensile test specimens. The enhanced tensile strength of 47.2 MPa and tensile modulus of 4.88 GPa were obtained for composites composed of 40 wt% hemp fiber (treated with a solution of 10% NaOH) and 3 wt% of MAPP. Beckermann et al. [32] reported that the maximum value of tensile strength (50.5 MPa) and tensile modulus (5.31 GPa) were found for composites containing 40 wt% of HF (treated with a solution of 5 wt% NaOH and 2 wt% Na_2_SO_3_) and 4 wt% of MAPP on PP matrix. Sain et al. [11] studied the effect of MAPP on the mechanical properties of natural fiber (among which HF is included)-reinforced PP composites. The optimum content of MAPP was found to be between 3–4 wt%. Hargitai et al. [33] investigated the effect of HF content on the mechanical properties of HF-PP nonwoven mat composites. They found that composites with HF content between 40 wt% and 50 wt%, displayed the optimum mechanical properties. Lu et al. [26] also observed that HF (treated with a solution of 5 wt% NaOH)-reinforced recycled HDPE composites with 40 wt% HF showed the highest tensile strength, tensile modulus, flexural strength and flexural modulus of 60.2 MPa, 2.57 GPa, 44.6 MPa, and 2.43 GPa, respectively. Torres et al. [34] used 3 wt% SA-treated sisal fiber-reinforced PE composites and reported an increase in the interfacial shear strength by 23% compared to untreated fiber composites.

The composite preparation procedure was as follows: (1) Pre-treatment of HF, PP and HDPE: HF was dried in an air oven (Carbolite Gero) at 140 °C (temperature based on TGA analysis) for 12 h, until a constant weight was reached. Thermal drying has been proved to be an effective method to reduce fibers’ moisture content and to improve the composite properties by improving the matrix-fiber interfacial adhesion [35,36]. PP and HDPE were also dried at 80 °C for 4 h, following the supplier’s recommendations. The presence of moisture prior to the melt blending or injection moulding process, might lead to poor processability and cause pores in the resultant materials as well as dimensional variations, which might negatively affect its mechanical properties [20,37]. (2) Composite preparation/pre-compound: all composite formulations were prepared based on weight percentages. According with the composite formulations in Table 4, PP or HDPE, HF, CA and SA were melt-blended using a Plastograph W50 (Brabender) under optimized processing conditions to prepare a pre-compound of materials in view of its subsequent processing. The torque as a function of time was monitored and recorded by the Brabender Mixer Program (WINMIX). About 1.5 Kg of each blended material was prepared for the injection process. (3) Composite preparation granulation: the resultant blended formulations were allowed to cool down to room temperature and then granulated using a granulator (Hellweg). (4) Injection moulding of test specimens: before the injection process the granulates were dried at 80 °C for 12 h in an air oven. The granulates were them injection moulded, into ISO standard dumbbell type 1A, ISO 3167—multipurpose test specimen [38], on a 220/150 E system (Arburg). PP and HDPE were also injection moulded into dumbbell type 1A specimens, in order to compare the mechanical and physical properties of both virgin and composite materials. The specifications such as setting parameters of the machine and the moulding conditions of the injected moulded specimens are listed in Table 5.

(5) Test specimen machining: the specimens for flexural tests, with length = 80 mm, width = 10 mm and thickness = 4 mm, were machined from the central portion of dumbbell type A1 specimen, according to ISO 178 [39] and ISO 179 [40].

### 2.3. Differential Scanning Calorimetry (DSC) and Thermal Gravimetric (TGA) Analyses

A Setsys instrumenr (Setaram; TGA sensibility of 0.1 mg) was the equipment used for both DSC and TGA analyses. DSC and TGA tests were performed on 8 to 10 mg of solid granulates of PP and HDPE at a heating rate of 3 °C/min. The solid granulates were heated from room temperature to 250 °C under synthetic air (O_2_ + N_2_), in order to characterize the polymer’s melting temperature and weight changes associate to polymer’s decomposition. Also, TGA was conducted to determine the thermal decomposition of the HF at a heating rate of 10 °C/min in the temperature range between room temperature and 500 °C under synthetic air (O_2_ + N_2_). The weight loss and its derivative were recorded as a function of temperature.

### 2.4. Water Absorption

Water absorption studies were conducted according to ISO 62:2008 [41]. The experimental method to determine the water absorption behaviour was the following: (1) twelve flexural test specimens of each composite formulation (Table 4) were dried to constant weight in an air oven at 50 °C for 24 h. For water absorption determination only three specimens of each composite formulation were evaluated (2) The specimens were cooled in a desiccator and (3) weighed using a MS204S/01 analytical balance (Mettler Toledo, 0.0001 g), after which (4) the specimens were immersed in distilled water at 23 °C. (5) At a designated time, the specimens were taken out from the water one at time, wiped with a dry cloth towel to remove the surface water, weighted and then (6) replaced in the distilled water. The amount of water absorbed by the specimens were measured (weight gain) every 24 h for 7 days and every 1 weeks for 2 months (1512 h), period of time in which the water equilibrium was reached. By using the weight gain data (average values of three weighings) with respect to time, the percentage of water absorbed was calculated and plotted versus the square root of time. The percentage of water absorption $W\;\left(\%\right)$ was calculated by the weight difference between the specimens immersed in water and the dried specimens, according to the following Equation (1):

(1) $$W\;\left(\%\right)=\frac{W_{t}-W_{0}}{W_{0}}\times 100$$

where $W_{t}$ is the weight of the specimen at time t, and $W_{0}$ is the initial weight of the specimen (at t=0). The equilibrium water content was determined as an averaged value of three consecutive weighings, which averages less than 1% of the total increase in weight [41].

### 2.5. Flexural Tests

Flexural tests were performed following the ISO 178:2003 [39]. Flexural properties were measured under a three-point bending loading with a span of 64 mm, with a nominal span/thickness of 16, using an Autograph AG-15 electromechanical testing machine (Shimadzu) with a 10 KN load cell and at a crosshead speed of 2 mm/min. The tests were carried out at an air temperature of 23 ± 5 °C and relative humidity of 50 ± 5%, which was the same environment in which all specimens were conditioned for 88 h, according to ISO 291 [42]. Five specimens, before aging (initial condition), of each composite formulation (Table 4) were tested and the average values of flexural modulus $\left(E_{f}\right)$ and flexural strength $\left(\sigma_{fm}\right)$ as well as the standard deviations are reported. In addition, flexural tests were also performed on wet specimens at the end of immersion time (1512 h) and on re-dried aged specimens. Redried aged specimens are the wet specimens which were dried at 80 °C for 48 h in an air oven, until reaching constant weight. The composite specimens were tested to failure, whereas PP and HDPE specimens were only tested to flexural strength $\sigma_{fm}$ due to excessive strain.

### 2.6. Scanning Electron Microscopy (SEM)

To characterize the morphology, evaluate the state of adhesion/dispersion of the fibers in the matrix and the effects of water absorption on the microstructure of composites SEM observations of flexural fracture surfaces of specimens before aging and re-dried aged specimens, were performed using a Merlin Gemini 2 instrument (Zeiss) The fractures portions of the specimens were cut and sputter-coated with a fine layer of gold over the surface uniformly for examination.

## 3. Results and Discussion

### 3.1. DSC and TGA Analyses

**Figure 1:**
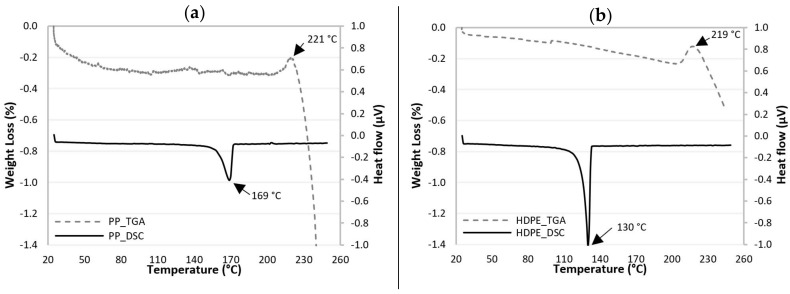
TGA and DSC curves for (**a**) PP and (**b**) HDPE.

Figure 1a,b show the DSC and TGA curves as a function of temperature, for PP and HDPE, respectively. The DSC curve of PP show an endothermic melting peak at 169 °C. The TGA curve shows that the onset of PP degradation occurs at around 221 °C, while the DSC curve for HDPE exhibits an endothermic melting peak at 130 °C and the TGA curve show that the degradation begins at around 219 °C.

**Figure 2:**
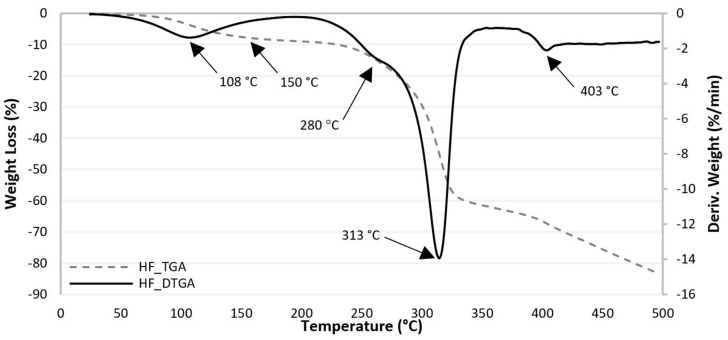
TGA and DTGA curves for HF.

Figure 2 shows the TGA and differential weight loss (DTGA) curves for hemp fibers with the increase of temperature. Table 6 lists the decomposition temperature peaks of HF corresponding to a weight loss at 500 °C.

The TGA curve of HF shows that thermal degradation starts at around 150–200 °C and becomes rapid at 280 °C. The thermal degradation of HF is a four-stage process. The DTGA curve show an initial peak at 108 °C (weight loss of 5%), which is due to the loss of water and is the first stage. According to Wielage et al. [43] hemicellulose is generally thought to decompose first, followed by cellulose and lignin. Therefore, the second degradation stage observed between 230–290 °C (weight loss 13%), with the maximum decomposition rate at 280 °C is associated with the degradation of hemicellulose. The third degradation stage, which is attributed to the degradation of cellulose, occurs at temperatures between 290–337 °C (weight loss of 36%), with the maximum decomposition rate at 313 °C. Lignin is the most difficult component to decompose and its decomposition occurred slowly, with a maximum decomposition rate at 403 °C (weight loss 67%) [13,37,44].

### 3.2. Torque Rheometry Analysis

**Figure 3:**
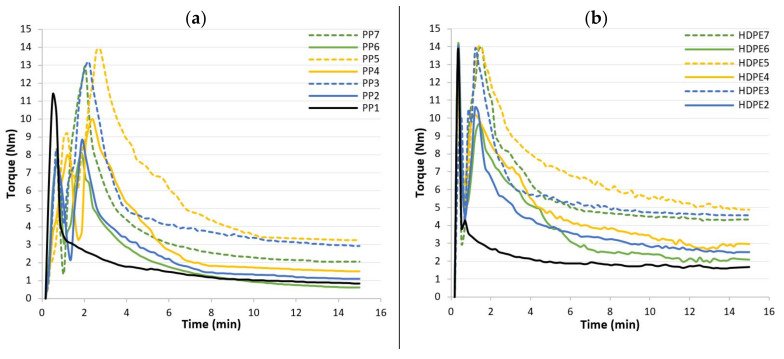
Torque versus time curves for (**a**) composite formulations based on a PP matrix and for (**b**) composite formulations based on a HDPE matrix.

Mixer torque rheometry experiments were used to investigate the rheological behaviour of composite formulations (Table 4) and also to blend the amount of material required to the injection moulding processes. Torque–rheometer plots as a function of blending time for composites based on PP and HDPE matrices are shown in Figure 3a,b, respectively, while Table 7 lists the steady state values obtained at 15 min of mixing.

The two-stage feeding are clearly shown in the rheograms: (1) From 0 to 2 min processing time, PP or HDPE, CA and SA are first mixed in the mixing chamber, according with the composite formulations (Table 4). (2) At the end of 2 min, the required quantity of HF is added into the mixing chamber and is continually mixed for 13 min, until homogenization. The torque values increase due to material resistance against the mechanical shear from the counter rotating rotors and with time the torque decreases and a steady state condition is obtained. The mixing parameters were optimized by varying the mixing time, rotor speed and chamber temperature. The objective was twofold: (1) to reduce the homogenization time to avoid PP, HDPE and HF thermal degradation and (2) to decrease the values of torque. The lower the torque, the easier is the mould filling process. Therefore, a mixing time of 15 min, a rotor speed of 60 rpm and a mixing temperature of 190 °C were found to be the optimum mixing conditions. For instance, the torque in the steady state for all formulations, was reached between 8 and 15 min, according to Figure 3. In all cases, an increase of the steady state values (Table 7) was found with increasing of HF content 20 wt% to 40 wt% and with the addition of CA, whereas the torque decreases with the addition of SA. The mobility of polymer chains is reduced due to addition of HF, consequently the melt viscosity is increased. The slight increase of torque with the addition of CA might be due to the occurrence of chemical reactions between the modified polymer with maleic anhydride-grafted in the chain and the hydroxyl groups of the hemp fibres. During grafting, the hydroxyl groups of hemp fibers reacts with maleic anhydride functional groups to form ester bonds. On the other hand, the addition of SA improves processability by reducing both shear viscosity and torque of the molten formulations. The steady state values at 15 min (Table 7) are higher for composites based on HDPE matrix than those of composites formulations based on PP, which might be due to the HDPE lower melt flow index (Table 3) and consequently lower fluidity.

### 3.3. Water Absorption Kinetics 

**Figure 4:**
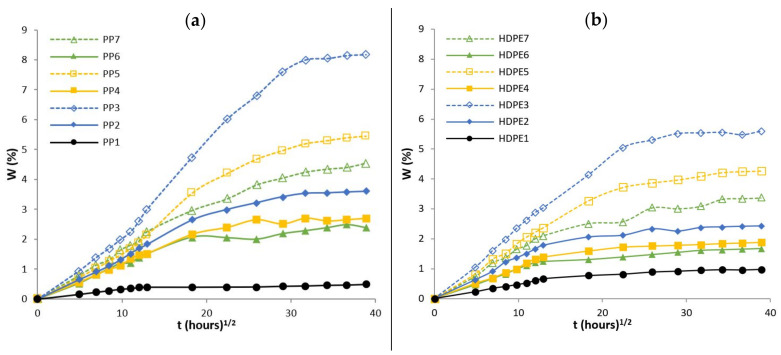
Water absorption curves of (**a**) PP and composites based on a PP matrix and (**b**) HDPE and composites based on a HDPE matrix.

Water absorption is the main parameter affecting the mechanical properties and dimensional stability of composites based on natural fibers. Water penetration into composites happens mainly due to a diffusion mechanism. The diffusion mechanism is characterized by the ability of water molecules to move among the micro gaps between polymer segments. The other two mechanisms are: (1) capillary flow of water molecules along the matrix-fiber interface, which is important when the matrix-fiber interfacial adhesion is poor or when the debonding of the matrix-fiber interface has begun and (2) transport of water molecules by microcracks in the matrix induced by swelling of fibers, which includes the flow and storage of water in the microcracks or in small channels in the composite [12,45]. Experimentally water absorption curves for PP and composites based on PP matrix as well as for HDPE and composites based on HDPE matrix are shown in Figure 4a,b, respectively. The percentage of $W\;\left(\%\right)$, is plotted against square root of time in hours $t\;{\left(\mathrm{hours}\right)}^{1/2}$.

Each data point represents the average values of three specimens. It is observed that the water absorption curves exhibits, two distinct features: (1) the $W\;\left(\%\right)$ increases rapidly and linearly with $t\;{\left(\mathrm{hours}\right)}^{1/2}$ until, (2) a plateau region is achieved (equilibrium water content), obeying to Fickian diffusion. The equilibrium water content of PP, HDPE and respective composites are summarized in Table 8.

The hydrophilic character of hemp fibres results in an increase of water absorption in the composites and therefore, a higher fiber content leads to: (1) an increase of the interfacial area (capillary effect), (2) an increase of the initial rate of water absorption and (3) a higher amount of water at the equilibrium. Also, hemp fibers have internal pathways for water transport (lumen) which most likely led to an increase of water absorption [25].

**Figure 5:**
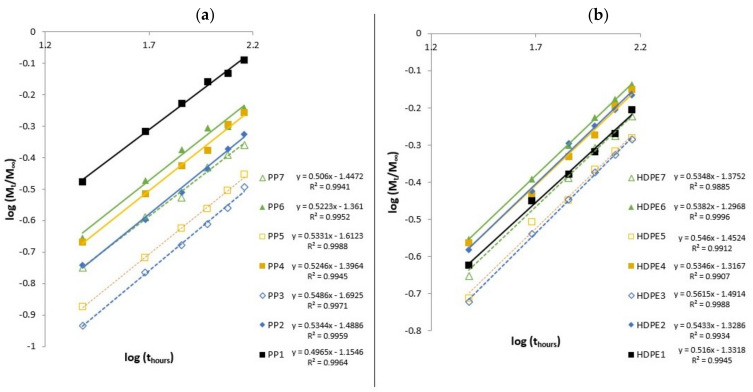
Diffusion parameters for (**a**) PP and composites based on a PP matrix and for (**b**) HDPE and composites based on a HDPE matrix.

The water absorption of composites based on HDPE matrix were lower than those of composites based on PP. Composites with CA and CA + SA showed a decrease in the initial rate of water absorption as well as a decrease of amount of water at equilibrium. From values analysis on $M_{\infty }$ of composites based on PP matrix (Table 8), for PP2 (PP + 20 wt% HF) and PP6 (PP + CA + SA + 20 wt% HF) and for PP3 (PP + 40 wt% HF) and PP7 (PP + CA + SA + 40 wt% HF), it is observed a decrease on the $M_{\infty }$ values by about 32.3% and 45.4%, respectively. Composites based on a HDPE matrix also show a decrease in their $M_{\infty }$ values by about 31.3% for HDPE2 (HDPE + 20 wt% HF) and HDPE6 (HDPE + CA + SA + 20 wt% HF) as well as 39.6% for HDPE3 (HDPE + 40 wt% HF) and HDPE7 (HDPE + CA + SA + 40 wt% HF). The enhanced resistance to water absorption of composites due to the addition of CA + SA suggests that the interfacial adhesion between matrix and fiber was improved, as both the functional groups of maleic anhydride and the carboxyl groups of SA react with hydroxyl groups of hemp fiber, reducing the available polar groups on the hemp fiber’s surface [22]. Water absorption of PP and HDPE, by its hydrophobic nature is almost negligible 0.46% and 0.97%, respectively. Therefore, the matrix had little effect on the amount of water absorption. The analysis of the water diffusion mechanism and kinetics were performed based on the Fick’s theory. The diffusion behaviour in thermoplastic composites reinforced with natural fibers can be classified in three categories, depending on the relative rates of diffusion and polymer relaxation. In Case I or Fickian diffusion, the rate of diffusion is much lower than the rate of relaxation. The equilibrium is reached quickly and it is maintained with independence of time. Water diffusion in natural fibers reinforced thermoplastic composites usually follows Case I. Case II (and super Case II), the diffusion is rapid compared with the relaxation process. Case III, non-Fickian or anomalous diffusion, is an intermediate behaviour between Case I and Case II and occurs when the diffusion and relaxation are comparable. Theoretically these three cases can be shaped by the Equation (2). Equation (3) is derived from Equation (2) [46]:

(2) $$\frac{M_{t}}{M_{\infty }}=kt^{n}$$

and:

(3) $$\log\;\left(\frac{M_{t}}{M_{\infty }}\right)=\log\left(k\right)+n\log\left(t\right)$$

where $M_{t}$ is the water content at time t, $M_{\infty }$ is the water content at the equilibrium, k is a constant characteristic of the specimen, which provides an idea about the interaction of water with the material, while n can change depending on the mode of diffusion [12]. Depending on coefficient n the diffusion process can be classified as: Case I, or Fickian diffusion $\left(n=0.5\right)$ and pseudo-Fickian (n<0.5), while for Case II $\left(n=1\right)$ and super Case II (n>1). For non-Fickian or anomalous diffusion, Case III (0.5<n<1) [46]. The coefficients k and n were determined by linear regression analysis based on experimental data (Equation (3)), according with Figure 5a,b and are summarized in Table 9.

The absorption of water in hemp fiber composites approaches Fickian diffusion (Case I), as the values of n are very close to n=0.5. The coefficient k values, are lower for PP (PP1) and HDPE (HDPE1), while for composites with 40 wt% of fiber content, both PP3 and HDPE3, the k values are higher, which means a higher interaction between the water and composite materials [47]. Therefore, as the water absorption approaches towards Fickian diffusion, the diffusion coefficient D, can be calculated by using the following equation [10,48,49]:

(4) $$D=\pi {\left(\frac{\theta h}{4M_{\infty }}\right)}^{2}$$

where θ is the initial and linear portion of $W\;\left(\%\right)$ versus $t\;{\left(hours\right)}^{1/2}$ plot and h is the initial thickness of the specimen [48,49]. The calculated diffusion coefficient D, are in Table 10, for PP and composites based on PP matrix and for HDPE and composites based on HDPE matrix, respectively. It is observed that the diffusion coefficient increases with fibre content and falls on the order of 10^−6^ $\left({\mathrm{mm}}^{2}/s\right)$, which is in agreement with other authors’ reports [46,47,48]. Due to the fibers’ inherent hydrophilic character, the water penetration into composites is favoured and the expanded interfacial area (capillary effect), benefits the transport of water through the matrix-fiber interface, leading to a greater diffusivity. Composites without CA and CA + SA showed higher diffusion coefficients, while for composites with CA and CA + SA the diffusion coefficients are lower, since there are more hydrophilic groups which are blocked by the coupling effect. Also, PP composites show slightly higher values of diffusion coefficient than HDPE composites. It appears that composites based on HDPE matrix show an improved behaviour against water absorption, since their diffusion coefficient values are generally the lowest. The permeability of water molecules through the composite depends on the water absorbed by the fiber. The permeability coefficient P, is given by Equation (5) [10,48,49]:

(5) P=D×S

where S is the absorption coefficient S that can be calculated by Equation (6) [10,48,49]:

(6) $$S=M_{\infty }/W_{0}$$

The values of P and S of PP and HDPE composites are given in Table 10. It is clear from Table 10 that the permeability coefficient increases with increasing fiber content and decreases due to the addition of CA and SA.

The experimental results were compared according to the model developed [50,51] for one-dimensional water absorption, following Fickian diffusion, in which the normalized absorption $\left(M_{t}/M_{\infty }>0.6\right)$, is expressed as function of D and h and is described by the following equation:

(7) $$\frac{M_{t}}{M_{\infty }}=1-\frac{8}{\pi^{2}}\;\overset{\infty }{\underset{n=0}{\sum }}\frac{1}{{\left(2n+1\right)}^{2}}\exp\left(-\frac{D{\left(2n+1\right)}^{2}\pi^{2}t}{h^{2}}\right)$$

**Figure 6:**
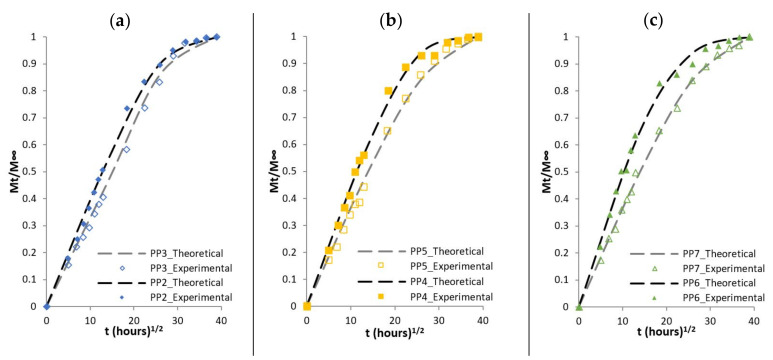
$M_{t}/M_{\infty }\;\mathrm{versus}\;t{\left(\mathrm{hours}\right)}^{1/2}$ for (**a**) PP2 and PP3, (**b**) PP4 and PP5 and (**c**) PP6 and PP7. Comparison of the Experimental data (dots) and theoretical curves (dashed lines).

**Figure 7:**
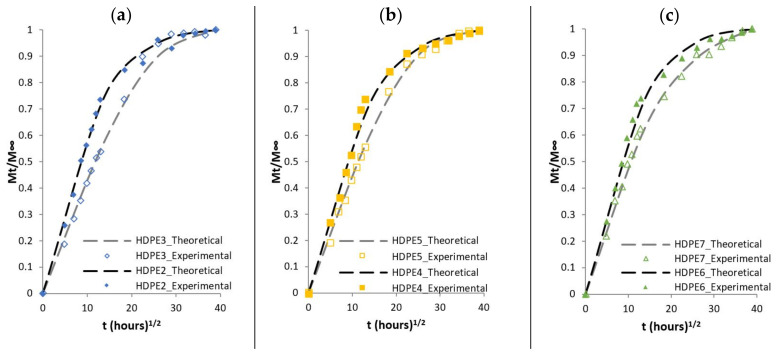
$M_{t}/M_{\infty }\;\mathrm{versus}\;t{\left(\mathrm{hours}\right)}^{1/2}$ for (**a**) HDPE2 and HDPE 3, (**b**) HDPE 4 and HDPE 5 and (**c**) HDPE 6 and HDPE 7. Experimental data (dots) versus theoretical curves (dashed lines).

The following comparison aims to check whether the theoretical model and the parameters determined above, can reproduce the response of the experimental diffusion results. Experimental and theoretical curves are compared for composites based on PP matrix as well as for composites based on HDPE matrix in Figure 6 and Figure 7, respectively. The dashed lines represent theoretical results while the dots represent the results from the experimental results. The theoretical results capture the initial slope (linear initial stage) up to ±30%, however beyond the initial stage, the concave curves to the abscissa, does not exactly fit the experimental results, in particularly for composites materials such as PP4, PP5 and PP6 or HDPE3, HDPE4 and HDPE6.

### 3.4. Effects of Water Absorption on the Flexural Properties

**Figure 8:**
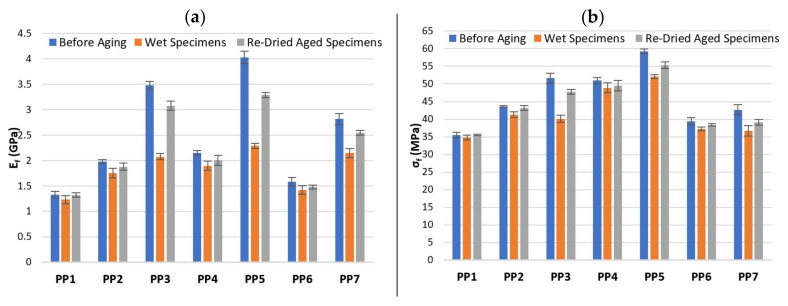
Effect of water absorption on (**a**) $E_{f}$ and (**b**) $\sigma_{fm}$ for PP and composites based on a PP matrix.

**Figure 9:**
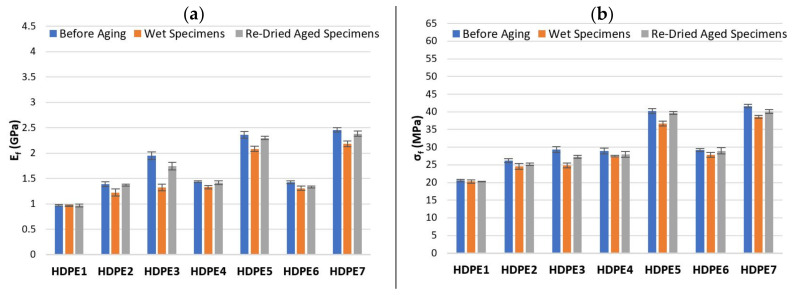
Effect of water absorption on (**a**) $E_{f}$ and (**b**) $\sigma_{fm}$ for HDPE and composites based on a HDPE matrix.

Flexural tests were performed on specimens before aging (initial condition), wet specimens at the end of immersion time (1512 h) and on re-dried aged specimens. The effect of water absorption on flexural modulus $\left(E_{f}\right)$ and flexural strength $\left(\sigma_{fm}\right)$ of PP and composites based on PP matrix as well as HDPE and composites based on HDPE matrix are shown in Figure 8 and Figure 9, respectively and the corresponding values are summarized in Table 11 and Table 12.

In the flexural tests, the load is transferred through the matrix-fiber interface and consequently the interfacial adhesion between matrix and hemp fiber largely determines the flexural mechanical properties of the composites. For specimens before aging, Figure 8 and Figure 9 shows that the flexural mechanical properties of composites based on HDPE matrix are lower than composites based on PP, moreover, the $E_{f}\;and\;\sigma_{fm}$ increases with both, fiber content and addition of CA. Although, composites with CA + SA (PP6, PP7 and HDPE6, HDPE7) show two distinct flexural behaviour: (1) HDPE6 and HDPE7 composites exhibited slightly higher values of $E_{f}\;and\;\sigma_{fm}$, whereas (2) PP6 and PP7 composites, both the values of $E_{f}\;and\;\sigma_{fm}$ decreased considerably with respect to CA composites (PP4 and PP5) by about 26.8% (PP4 to PP6), 30.3% (PP5 to PP7) for $E_{f}$ and by about 22.6% (PP4 to PP6), 28.1% (PP5 to PP7) for $\sigma_{fm}$. The addition of SA compromised the flexural mechanical properties of PP6 and PP7 composites. Similar mechanical behaviour is found on studies on cork particles reinforced PP composites in which MAPP and SA are used as coupling agents, by Fernandes et al. [22]. The results show that the tensile strength and tensile modulus decreases with the addition of SA by about 21.7% and 3.9% with respect to composites with MAPP. Dányádi et al. [52] observed in their experiments on PP composites reinforced with 20% wood flour, that the addition of SA led to a moderate reduction of tensile strength. Also, similar conclusions are found in the study performed by Stark [53] in which the tensile and flexural mechanical properties were slight reduced upon SA addition to a 40% wood-reinforced PP composite. According to Dalvag et al. [54] the decrease in tensile strength might be due to the plasticizing effect on the PP matrix resulting from the addition of SA, which can give rise to poorly bonded regions between matrix-fiber, leading to ineffective stress transfer. Nevertheless, some research studies [22,52,53] have reported a reduction of water absorption, improvements on homogeneity (better dispersion of the fiber throughout the matrix) and processability of composites with the addition of SA. Enhanced (Table 11 and Table 12) $E_{f}$ of 4.03 and 2.46 GPa, and $\sigma_{fm}$ of 59.31 and 41.64 MPa were obtained for PP5 (CA) and HDPE7 (CA + SA) composites composed of 40 wt% hemp fiber, respectively. The percentage increase of $E_{f}$ and $\sigma_{fm}$ of PP5 over PP1 and HDPE7 over HDPE1 are 205.3%, 67.4% and 152.5%, 102.5%, respectively. The improved flexural properties of PP5 and HDPE7 might be due to an increase in the interface adhesion between matrix-fiber, which allows a better and effective load transfer throughout the interface [48]. PP2, PP3, HDPE2 and HDPE3 composites exhibited low flexural mechanical properties, which might be due to the poor interaction between the hydrophilic HF and the hydrophobic matrices.

The observed effect of water absorption on the flexural properties, show the expected tendencies: (1) water absorption by the composites resulted in a decrease of $E_{f}$ and $\sigma_{fm}$ in both situations: at the end of immersion time and on re-dried aged specimens, while (2) the flexural properties of PP and HDPE remains unchanged with immersion time in water. Despite the decreased of $E_{f}$ and $\sigma_{fm}$ due to water absorption, composites based on HDPE matrix showed, in general, a lower reduction of $E_{f}$ and $\sigma_{fm}$ than composites based on PP. The percentage reduction in $E_{f}$ and $\sigma_{fm}$ of composites based on PP matrix (Table 11), for specimens before aging over wet specimens are found to be, 11.5% and 5.5% for PP2, 40.3% and 22.3% for PP3, 11.8% and 4.1% for PP4, 43.1% and 12.2% for PP5, 9.9% and 5.7% for PP6, 23.7% and 13.8% for PP7, respectively. Composites based on HDPE matrix (Table 12), also show a decrease on the $E_{f}$ and $\sigma_{fm}$ by about 11.7% and 6.2% for HDPE2, 32.2% and 15.3% for HDPE3, 7.4% and 4.9% for HDPE4, 11.9% and 8.9% for HDPE5, 8.5% and 4.6% for HDPE6, 11.3% and 7.3% for HDPE7, respectively. Therefore, the decrease of $E_{f}$ and $\sigma_{fm}$ in the composites might be attributed to the effect of water molecules, which change the structure and properties of hemp fibers and the interface between matrix-fiber, due to the formation of hydrogen bonds between the water molecules and cellulose fibers. Also, water absorption leads to fiber swelling, formation of cracks and debonding of the matrix-fiber interface, affecting the dimensional stability of the composites and the mechanical properties [48]. This effect is particularly greater for the composites with higher amount of fiber content.

The effect of water absorption on the flexural properties of re-dried aged specimens were also evaluated and compared to specimens before aging. From Table 11 and Table 12 is observed that the $E_{f}$ and $\sigma_{fm}$ values were not completely recovered. Although, the decrease in $E_{f}$ and $\sigma_{fm}$ values are relatively lower compared to wet specimens. Therefore, the absorption of water by the composites resulted in permanent damage after long-term aging. Moreover, besides the deterioration of composite’s flexural properties due to water absorption, the dissolution of low molecular weight soluble polymers from HF such as short chain hemicellulose, lignin and pectin, might lead to material loss [36] as well as colour/appearance change on the surface of re-dried aged specimens. Some material loss was observed by comparing the weight difference between re-dried aged specimens ($W_{\mathrm{re}-\mathrm{dried}}$) and $W_{0}$ and the values are summarized in Table 13. It is observed that the weight loss increases with increasing of fiber content.

**Figure 10:**
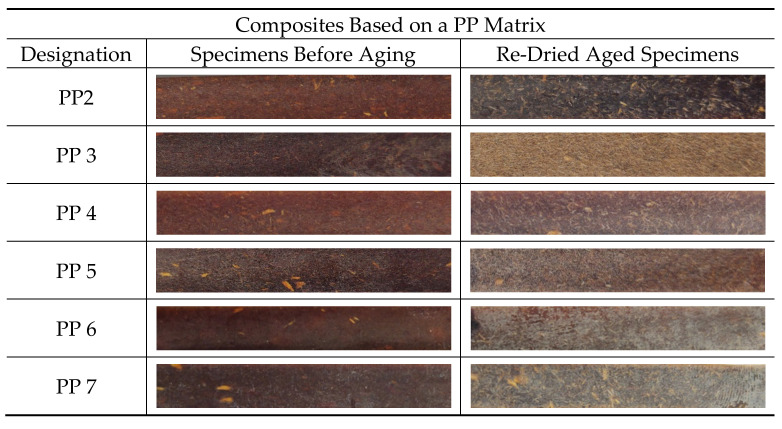
Colour change on re-dried aged specimens. Composites based on a PP matrix.

**Figure 11:**
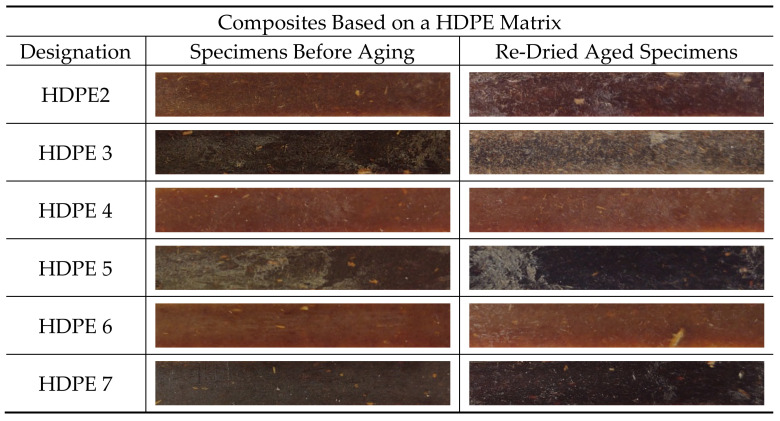
Colour change on re-dried aged specimens. Composites based on a HDPE matrix.

Also, the influence of water absorption on the colour change on the surface of re-dried aged specimens was analysed visually. The discoloration of thermoplastic composites reinforced with natural fibers is a disadvantage for outdoor applications, because the changes in appearance can have a negative impact on the aesthetic qualities of materials and they usually precede changes in mechanical properties [55]. Figure 10 and Figure 11 show the colour changes on the re-dried aged specimens. It can be seen that all re-dried aged specimens show permanent colour fading after 1512 h of water immersion.

Composites based on HDPE matrix, namely HDPE4, HDPE5, HDPE6 and HDPE7, show better colour retention compared to composites based on PP matrix and appear to maintain their original colour. The colour change in natural fibers is mainly attributed to delignification (lignin degradation or separation) and formation of carboxyl groups [56,57]. Therefore, the addition of CA or CA + SA on composites based on HDPE matrix, seems to influence the colour stability due to loss reduction of water-soluble matter (Table 13).

### 3.5. SEM Analyses

**Figure 12:**
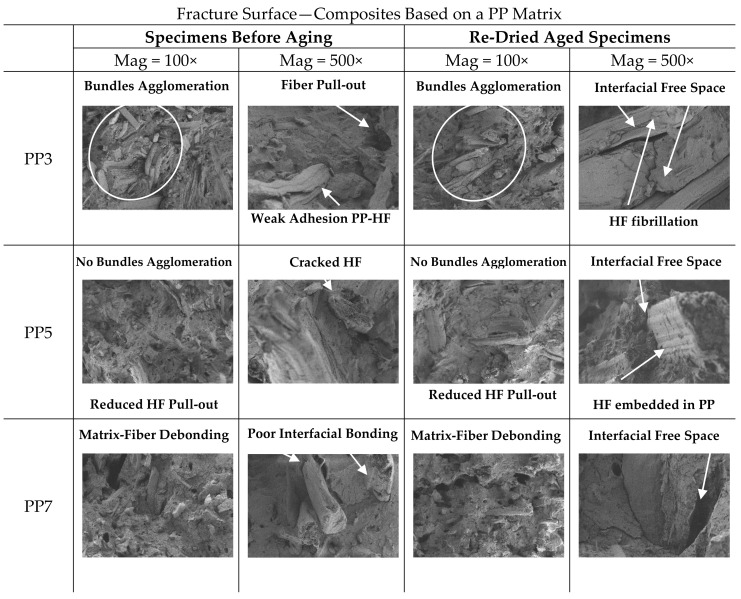
SEM fracture surfaces for PP3, PP5 and PP7 before aging and re-dried aged specimens.

**Figure 13:**
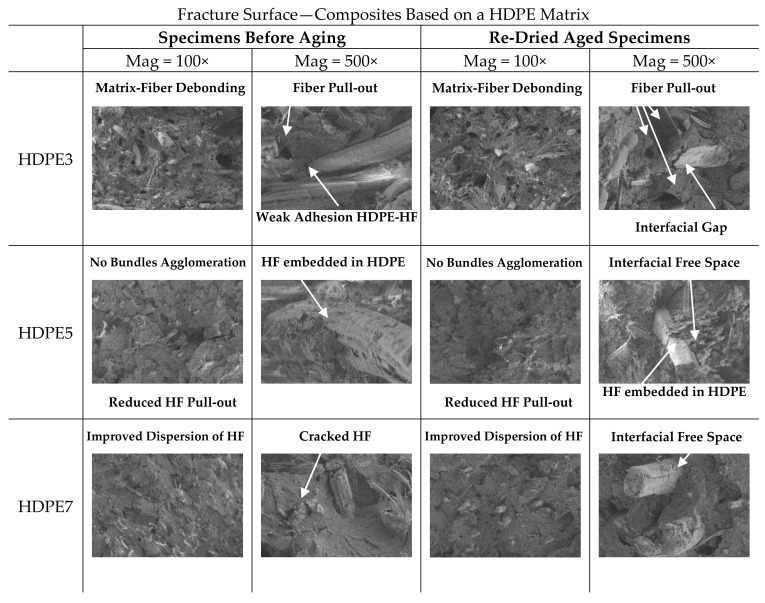
SEM fracture surfaces for HDPE3, HDPE5 and HDPE7 before aging and re-dried aged specimens.

PP3, PP5, PP7 and HDPE3, HDPE5, HDPE7, experienced full section fracture under bending loading. Fracture surfaces of representative specimens before aging and re-dried aged specimens (PP3, PP5, PP7 and HDPE3, HDPE5, HDPE7) are displayed in Figure 12 and Figure 13.

Qualitative SEM observations of PP3 and HDPE3, fractured surfaces at two magnifications, specimens before aging, show poor interaction between the fibre and matrix and several fiber agglomerates due to the weak interfacial bonding and poor dispersion of the fibers within the matrix. The phenomenon of fiber pull-out from the matrix, occurred to a greater extent causing the failure of composite material, suggesting that the failure mechanism could have resulted from matrix-fiber debonding. Also, due to absence of CA and SA, the fibers tend to agglomerate into bundles and become unevenly distributed throughout the matrix. On the other hand, SEM micrographs of PP5 and HDPE5, composites with CA, specimens before aging, exhibit better interfacial adhesion, which is evident from Figure 12 (PP5) and Figure 13 (HDPE5). It is observed that the phenomenon of fiber pull-out is reduced and there is a better dispersion of fibers in the matrix. In addition, SEM micrographs shows that the hemp fibers are embedded in thermoplastic matrices, with cracked hemp fibers, while for PP3 and HDPE3 the hemp fibers appear smooth and clean, with non-evidence of PP or HDPE adhering to the fiber surfaces. Therefore, both the wettability of hemp fibers in the matrix and the interfacial shear strength at the interface matrix-fiber are improved with the addition of CA, which also explains the improvements in the flexural properties and water resistance of composites. SEM micrographs of PP7 and HDPE7 Figure 12 (PP7) and Figure 13 (HDPE7), composites with CA + SA, specimens before aging, show two distinct fracture surfaces: (1) SEM micrographs of HDPE7, shows the better dispersion, strong interfacial bonding, smother surface and less micro voids content. Therefore, the enhanced morphological properties also reflect the enhanced flexural properties and water resistance of composites, while (2) SEM micrographs of PP7, shows that the addition of SA had a negative impact on the morphological properties. It is observed micro voids due to fiber pull-out, rough surfaces and fiber bundles. The addition of SA compromised the matrix-fiber interfacial adhesion, which was reflected in a considerable decrease in the flexural mechanical properties.

The effects of water absorption on the microstructure of re-dried aged specimens are also shown in Figure 12 and Figure 13. Generally, in re-dried aged specimens aged fibers are observed, surrounded by micro-cracks in the matrix (interfacial free space), where the loss of adhesion between fiber and matrix led to the deterioration of the flexural mechanical properties. Aged HF due to water absorption is confirmed by the presence of fibrillation, Figure 12 (PP3). Damage modes such as matrix-fiber interfacial debonding with holes left where fibres have been pulled out during flexural testing, are visible in the fractured surface. It can be assumed that fiber swelling in the transverse direction induces hoop stresses in the matrix, leading to plastic strain and micro-cracks. After drying, the shrinkage of fibres results in free space at the interface between matrix-fiber, which might contribute to fibre-matrix decohesion and hence weaken the interface and decreasing the flexural properties of the composites. This kind of damage has been observed also by other authors [14,18,21,58].

## 4. Conclusions

Both PP and HDPE short hemp fiber-reinforced composites were injection moulded. The effect of CA, SA and water absorption on the physical and flexural properties of the resulting composites were investigated. The following main conclusions are drawn:

– The water absorption behaviour of composites showed a Fickian mode of diffusion, where the kinetics parameters are influenced by the polymeric matrix, fiber content and matrix-fiber interfacial adhesion (CA + SA).
– The addition of CA + SA to composites resulted in a decrease of: (1) initial rate of water absorption, (2) amount of water at equilibrium, (3) diffusion coefficient, which falls on the order of 10^−6^ $\left({\mathrm{mm}}^{2}/s\right)$ and (4) permeability coefficient, while both the diffusion and permeability coefficients increase with increasing fiber content in the composites.
– PP composites show slightly higher values of water absorption and diffusion coefficients than HDPE composites.
– Composites experienced weight loss as well as colour fading after 1512 h of water immersion, however composites based on a HDPE matrix, namely HDPE4, HDPE5, HDPE6 and HDPE7, showed better colour retention. The addition of CA or CA + SA, positively influenced the colour stability of composites based on a HDPE matrix.
– Flexural mechanical property measurments for specimens before aging showed that enhanced $E_{f}$ of 4.03 and 2.46 GPa, and $\sigma_{fm}$ of 59.31 and 41.64 MPa were obtained for PP5 (CA) and HDPE7 (CA+SA) composites composed of 40 wt% hemp fiber, respectively. The improved mechanical properties are due to a better interfacial adhesion between the matrix and fiber and better dispersion of the fibers within the matrix, as confirmed by SEM micrographs.
– The addition of SA compromised the flexural mechanical properties of PP6 and PP7 composites, which might be attributed to a possible plasticizing effect on the PP matrix [54].
– The flexural mechanical properties of composites based on a HDPE matrix are lower than those of composites based on PP.
– Water absorption by the composites resulted in a decrease of $E_{f}$ and $\sigma_{fm}$ in both situations: at the end of immersion time and on re-dried aged specimens. Therefore, water absorption by the composites resulted in a permanent damage in the interfacial bonding between matrix-fiber, after log-term aging, which also was confirmed by SEM.
– Despite the decrease of $E_{f}$ and $\sigma_{fm}$ due to water absorption, composites based on a HDPE matrix showed lower reduction of $E_{f}$ and $\sigma_{fm}$ than composites based on PP.

Short hemp fibers reinforced PP or HDPE composites can be used for many indoor applications, such as decking, however, for outdoor applications under extreme environmental conditions such as humidity, their use should be greatly restricted due to their poor water resistance and dimensional stability (swelling). The long-term exposure to extreme humidity environment can lead to a negative impact on aesthetics qualities of composites as well as on its mechanical properties.

## Figures and Tables

**Table 1:** Literature review on hemp fiber reinforced polypropylene composites, based on injection mould production.

Materials	Composite Manufacturing	Research Achievements	Ref.
– Polypropylene: PP 6331, melt flow index, 14 g/10 min (230 °C/2.16 kg) and density, 0.90 g/cm^3^; – Short hemp fibers: length 12 mm; – Short E-glass fibers: length 6 mm; – Coupling agent: Orevac^®^- CA100, 10 g/10 min (190 °C/0.325 kg) and density, 0.905 g/cm^3^.	– (1) Materials were melt blended; – (2) Blended formulations were granulated; – (3) Granulates were them injection moulded at 200 °C.	– Fickian mode of water diffusion was observed for all composites immersed in distilled water at 40 °C. – The equilibrium of water content is temperature-independent, while the diffusion coefficient tends to increase as temperature increases. – Fiber hybridization reduced the water absorption. – The effect of water absorption on flexural mechanical properties of hybrid composites, showed a reduction in tensile modulus and tensile strength in both: wet specimens and in re-dried aged specimens.	[12]
– Polypropylene: Isplen PP 090 G2M, melt flow index, 35 g/10 min (230 °C/2.16 kg) and density, 0.90 g/cm^3^; – Short hemp fibers: length 10 mm; – Coupling agent: Epolene G3015 and density, 0.913 g/cm^3^.	– (1) Materials were mixed in a two-roll mill; – (2) Blended formulations were cut down to pellets; – (3) Pellets were injection moulded at 190 °C (nozzle temperature).	– Strong interfacial adhesion between hemp fibers and polypropylene were found with the addition of 4 wt% maleic anhydride poly(propylene) (MAPP). – The maximum value of tensile strength (48.8 MPa), tensile modulus (3.5 GPa), flexural modulus (58.6 MPa) and flexural modulus (4.3 GPa) were found for composite containing 40 wt% of hemp fiber (treated with a solution of 5 wt% NaOH and 2 wt% Na_2_SO_3_) and 4 wt% of MAPP on PP matrix.	[2]
– Polypropylene: Icorene^®^ PP CO14RM, melt flow index, 13 g/10 min (230 °C/2.16 kg) and density, 0.90 g/cm^3^; – Alkali treated hemp fibers: 10% NaOH solution at 160 °C for 45 min and length 1–3 and 10 mm; – Coupling agent: A-C 950P and density, 0.93 g/cm^3^.	– (1) Materials were compounded in a twin-screw extruder; – (2) Blended formulations were granulated into pellets; – (3) Pellets were injection moulded.	– Mechanical properties of composites were enhanced with 40 wt% hemp fiber (treated with a solution of 10% NaOH) and 3 wt% of MAPP. The maximum value of tensile strength and tensile modulus found were 47.2 MPa and 4.88 GPa, respectively.	[16]
– Polypropylene: melt flow index, 35 g/10 min (230 °C/2.16 kg) and density, 0.90 g/cm^3^; – Short hemp fiber: length 1 mm; – Coupling agent (not specified).	– (1) Materials were compounded in a thermokinetic mixer; – (2) injection moulded.	– The effect of temperature on mechanical properties (flexural, tensile and impact tests) of 25 wt% and 40 wt% hemp fiber reinforced polypropylene composites were evaluated. – The effect of temperature on mechanical properties showed that the impact resistance is temperature-independent, while flexural and tensile properties are strongly affected.	[24]

**Table 2:** Literature review on hemp fiber reinforced polyethylene composites, based on injection mould production.

Materials	Composite Manufacturing	Research Achievements	Ref.
– Polyethylene: Dow TM 12450N Health, melt flow index, 12 g/10 min (230 °C/2.16 kg) and density, 0.95 g/cm^3^; – Short hemp fibers; – Coupling agents: (1) Epolene C-26 and (2) DupontTM Fusabond WPC-576D.	– (1) composites were compound in twin-screw extruder; – (2) Cut in small pellets; – (3) The pellets were injection moulded at 190 °C.	– The addition of coupling agent on composites resulted in a decrease of water absorption and reduction of flexural yield strength.	[17]
– Polyethylene: AT 418, melt flow index, 12 g/10 min (230 °C/2.16 kg) and density, 0.916 g/cm^3^; – Short hemp fibers and other five natural fibers; – Coupling agents: Fusabond MB265D.	– (1) composites were compound in twin-screw extruder; – (2) Cut in small pellets; – (3) The pellets were injection moulded.	– The tensile modulus increased and the strain at failure as well as the impact strength decreased with increasing of hemp fiber content in the composites. – At 40 wt% of hemp fiber, composites had a tensile strength of 12.2 MPa. – Coupling agent was found to significantly increase the mechanical performance and reduce water absorption.	[25]
– Polyethylene: recycled high density polyethylene, melt flow index, 0.45 g/10 min (190 °C/2.16 kg) and density, 0.98 g/cm^3^; – Alkali treated hemp fibers: 5% NaOH solution	– Composites were manufactured by using a single-screw extruder and injection moulding process.	– Composites with 40 wt% alkali treated hemp fiber had the best mechanical properties: tensile strength, tensile modulus, flexural strength and flexural modulus.	[26]

**Table 3:** ISPLEN^®^ PP 090 G2M [27], RIOPOL^®^ BI-56250 [28], Fusabond™ M603 [29], Acros Organics™ stearic acid 97% [30] and short hemp fibers [31], technical information.

Materials	Density (g/cm^3^)	Melt Flow Index	Flexural Modulus (MPa)	Melting Point (°C)	Processing Conditions (°C)
PP	0.905	35 g/10 min	1650	-	190 to 250
	(ISO 1183)	(230 °C, 2.16 kg) (ISO 1133)	(ISO 178)		
HDPE	0.956	25 g/10 min	1032	-	170 to 200
	(ASTM D792)	(190 °C, 2.16 kg) (ASTM D1238)	(ASTM D790)		
CA	0.94	25 g/10 min	-	108	260
	(ISO 1183)	(190 °C, 2.16 kg) (ISO 1133)		(DSC) (ISO 3146)	(Maximum processing temperature)
SA	0.84	-	-	67 to 69	-
HF	1.4–1.5	-	-	-	<200
	1.514 *				

* The average densities of short hemp fibers were determined by using MicroMeritics AccuPyc 1330 (helium pycnometer).

**Table 4:** Composite formulations.

Composites Based on PP Matrix			Composites Based on HDPE Matrix		
Designation	Composition	Ratio (wt%)	Designation	Composition	Ratio (wt%)
PP1	PP	100	HDPE1	HDPE	100
PP2	PP|HF	80|20	HDPE2	HDPE|HF	80|20
PP3	PP|HF	60|40	HDPE3	HDPE|HF	60|40
PP4	PP|CA|HF	77|3|20	HDPE4	HDPE|CA|HF	77|3|20
PP5	PP|CA|HF	57|3|40	HDPE5	HDPE|CA|HF	57|3|40
PP6	PP|CA|SA|HF	74|3|3|20	HDPE6	HDPE|CA|SA|HF	74|3|3|20
PP7	PP|CA|SA|HF	54|3|3|40	HDPE7	HDPE|CA|SA|HF	54|3|3|40

**Table 5:** Specifications and set parameters of the injected moulded specimens.

Parameters	First Injection (s)	Second Injection (s)	Mould Cooling (s)	Cycle Time (s)	Injection Pressure (MPa)	Nozzle Temperature (°C)	Mould Surface Temperature (°C)
PP	4	10	85	99	7.5	190	25
HDPE	5	15	85	115	7.5	190	25

**Table 6:** Decomposition temperature peaks of HF corresponding weight loss at 500 °C.

1st Peak (°C)	2nd Peak (°C)	3rd Peak (°C)	4th Peak (°C)	Total Weight Loss (%) at 500 °C
108	280	313	403	80

**Table 7:** Steady state values for all composite formulations at 15 min.

Composites Based on a PP Matrix			Composites Based on a HDPE Matrix		
Designation	Torque (Nm)	Time (min)	Designation	Torque (Nm)	Time (min)
PP1	0.84	15	HDPE1	1.68	15
PP2	1.12		HDPE2	2.51	
PP3	2.93		HDPE3	4.57	
PP4	1.52		HDPE4	2.97	
PP5	3.26		HDPE5	4.89	
PP6	0.62		HDPE6	2.09	
PP7	2.06		HDPE7	4.34	

**Table 8:** Equilibrium water content.

PP and Composites Based on PP Matrix		HDPE and Composites Based on HDPE Matrix	
Designation	Equilibrium Water Content (%), $M_{\infty }$	Designation	Equilibrium Water Content (%), $M_{\infty }$
PP1	0.4642	HDPE1	0.9726
PP2	3.5784	HDPE2	2.4120
PP3	8.1112	HDPE3	5.5389
PP4	2.6507	HDPE4	1.8639
PP5	5.3797	HDPE5	4.2454
PP6	2.4230	HDPE6	1.6580
PP7	4.4281	HDPE7	3.3467

**Table 9:** Equilibrium water content.

PP and Composites Based on a PP Matrix			HDPE and Composites Based on a HDPE Matrix		
Designation	n	$k\;\left(h^{2}\right)$	Designation	n	$k\;\left(h^{2}\right)$
PP1	0.4965	0.0317	HDPE1	0.5160	0.0492
PP2	0.5344	0.1395	HDPE2	0.5433	0.1398
PP3	0.5486	0.2115	HDPE3	0.5615	0.2424
PP4	0.5246	0.1213	HDPE4	0.5346	0.1085
PP5	0.5332	0.1559	HDPE5	0.5460	0.1875
PP6	0.5223	0.1165	HDPE6	0.5382	0.1015
PP7	0.5060	0.1631	HDPE7	0.5348	0.1666

**Table 10:** Diffusion, absorption and permeation coefficients values of PP and composites based on a PP matrix and a HDPE and composites based on HDPE matrix.

PP and Composites Based on a PP Matrix				HDPE and Composites Based on a HDPE Matrix			
Designation	D × 10^−6^ (mm^2^/s)	S	P × 10^−6^ (mm^2^/s)	Designation	D × 10^−6^ (mm^2^/s)	S	P × 10^−6^ (mm^2^/s)
PP1	0.6234	1.0048	6.2648	HDPE1	0.8238	1.0099	8.3286
PP2	1.8085	1.0366	1.8737	HDPE2	1.6348	1.0244	1.6748
PP3	2.7505	1.0816	2.9751	HDPE3	2.5402	1.0554	2.6809
PP4	1.7261	1.0269	1.7731	HDPE4	1.5051	1.0187	1.5332
PP5	2.4424	1.0544	2.5754	HDPE5	2.3542	1.0429	2.4551
PP6	1.6622	1.0238	1.7019	HDPE6	1.4464	1.0168	1.4704
PP7	2.2983	1.0455	2.4029	HDPE7	2.2184	1.0341	2.2943

**Table 11:** $E_{f}$ and $\sigma_{fm}$ values of PP and composites based on PP matrix.

PP and Composites Based on a PP Matrix						
Designation	$E_{f}\;(\mathrm{GPa})$			$\sigma_{fM}\;(\mathrm{MPa})$		
	Before Aging	Wet Specimens	Re-Dried Specimens	Before Aging	Wet Specimens	Re-Dried Specimens
PP1	1.3288 [0.6]	1.2325 [0.8]	1.3238 [0.4]	35.4438 [0.8]	34.8090 [0.6]	35.5375 [0.2]
PP2	1.9840 [0.4]	1.7551 [1.1]	1.8814 [0.7]	43.6163 [0.2]	41.2388 [0.8]	43.1575 [0.7]
PP3	3.4822 [0.7]	2.0806 [0.6]	3.0811 [0.9]	51.6638 [1.4]	40.1300 [1.1]	47.7725 [0.6]
PP4	2.1565 [0.4]	1.9012 [0.9]	2.0085 [1.1]	50.9625 [0.9]	48.8775 [1.4]	49.5225 [1.5]
PP5	4.0314 [1.2]	2.2927 [0.5]	3.2935 [0.5]	59.3050 [0.7]	52.0963 [0.6]	55.3413 [0.9]
PP6	1.5832 [0.9]	1.4269 [0.8]	1.4787 [0.6]	39.4625 [0.9]	37.2225 [0.5]	38.3775 [0.3]
PP7	2.8197 [1.1]	2.1511 [0.6]	2.5468 [0.5]	42.6825 [1.4]	36.6625 [1.5]	39.1275 [0.8]

**Table 12:** $E_{f}$ and $\sigma_{fm}$ values of HDPE and composites based on a HDPE matrix.

HDPE and Composites Based on a HDPE Matrix						
Designation	$E_{f}\;(\mathrm{GPa})$			$\sigma_{fM}\;(\mathrm{MPa})$		
	Before Aging	Wet Specimens	Re-Dried Specimens	Before Aging	Wet Specimens	Re-Dried Specimens
HDPE1	0.9743 [0.1]	0.9685 [0.1]	0.9724 [0.3]	20.5625 [0.3]	20.2950 [0.5]	20.2688 [0.1]
HDPE2	1.3910 [0.5]	1.2278 [0.7]	1.3743 [0.2]	26.1850 [0.6]	24.5663 [0.8]	25.1513 [0.4]
HDPE3	1.9535 [0.8]	1.3253 [0.7]	1.7443 [0.7]	29.3213 [0.8]	24.8288 [0.7]	27.2625 [0.4]
HDPE4	1.4386 [0.2]	1.3319 [0.3]	1.4187 [0.4]	28.8975 [0.8]	27.4863 [0.2]	28.0050 [0.8]
HDPE5	2.3659 [0.6]	2.0851 [0.5]	2.3012 [0.3]	40.2563 [0.7]	36.6750 [0.6]	39.6500 [0.4]
HDPE6	1.4291 [0.3]	1.3077 [0.4]	1.3350 [0.1]	29.1825 [0.4]	27.8330 [0.7]	28.9350 [0.9]
HDPE7	2.4597 [0.4]	2.1824 [0.6]	2.3838 [0.5]	41.6350 [0.5]	38.5889 [0.5]	40.1325 [0.5]

Note: Standard deviation [in square brackets].

**Table 13:** Lost weight (%) ($W_{\mathrm{re}-\mathrm{dried}}-W_{0}$) for all composites.

PP and Composites Based on a PP Matrix				HDPE and Composites Based on a HDPE Matrix			
Designation	$W_{0}\;(g)$	$W_{re-dried}\;(g)$	Lost weight (%)	Designation	$W_{0}\;(g)$	$W_{re-dried}\;(g)$	Lost weight (%)
PP2	7.4768	7.4690	0.1043	HDPE2	7.6783	7.6845	0.0803
PP3	8.2704	8.2035	0.8093	HDPE3	8.3468	8.3317	0.1809
PP4	7.5427	7.5389	0.0504	HDPE4	7.6595	7.6629	0.0448
PP5	8.4234	8.4139	0.1124	HDPE5	8.2850	8.2779	0.0861
PP6	7.3497	7.3387	0.1492	HDPE6	7.5671	7.5636	0.0467
PP7	8.1721	8.1557	0.2007	HDPE7	8.2640	8.2715	0.0912

## Data Availability

Data is available on demand from the corresponding author.
